# Effectiveness of different bathing methods on physiological indexes and behavioral status of preterm infants: a systematic review and meta-analysis

**DOI:** 10.1186/s12887-023-04280-y

**Published:** 2023-10-13

**Authors:** Xing Sun, Jiayi Xu, Ruhua Zhou, Beibei Liu, Zejuan Gu

**Affiliations:** 1 School of Nursing, Nanjing Medical University, 101 Longmian Road, Nanjing, 211166 China; 2 School of Nursing, Nanjing University of Chinese Medicine, 138 Xianlin Road, Nanjing, 210023 China; 3 The First Affiliated Hospital with Nanjing Medical University, 300 Guangzhou Road, Nanjing, 210029 China; 4 Women’s Hospital of Nanjing Medical University/Nanjing Maternity and Child Health Care Hospital, 123 Tianfei Road, Nanjing, 210004 China

**Keywords:** Preterm infant, Bath, Swaddle bath, Conventional tub bath, Sponge bath, Meta-analysis

## Abstract

**Background:**

Bath is an external stimulus for preterm infants. Currently, three methods are used for preterm infants to bath. It is important to choose the best way for them. The objective of this meta-analysis is to evaluate the effectiveness of different bath methods on physiological indexes and behavioral status of preterm infants.

**Methods:**

This systematic review was conducted according to the Preferred Reporting Items for Systematic Reviews and Meta-Analyses [PRISMA] guidelines and was registered in PROSPERO(CRD42022377657). PubMed, Embase, Cochrane Library, Web of Science, CINAHL, Sino Med, China National Knowledge Internet (CNKI) and Wan-Fang database were systematically searched for randomized controlled trials on the effects of different bath methods for preterm infants. The retrieval time was from the establishment of the database to February 2023. According to the inclusion and exclusion criteria, the literature was screened, quality evaluated and the data was extracted. Reman Version 5.4 was used for meta-analysis and Stata 16.0 software for publication bias Egger’s test.

**Results:**

A total of 11 RCTs with 828 preterm infants were included. The results of meta-analysis showed that the body temperature and oxygen saturation of preterm infants in the sponge bath group were lower than those in conventional tub bath group (SMD = -0.34; 95%CI -0.56 to -0.12; I^2^ = 0;* p* < 0.01), (MD = -0.39; 95%CI -0.76 to -0.02; I^2^ = 39%;* p* = 0.04), while the heart rates were higher than those in conventional tub bath group(MD = 5.90; 95%CI 0.44 to 11.35; I^2^ = 61%;* p* = 0.03). Preterm infant’s body temperature and blood oxygen saturation of in swaddle bath group were higher than those in conventional tub bath group (MD = 0.18; 95%CI 0.05 to 0.30; I^2^ = 88%;* p* < 0.01), (MD = 1.11; 95%CI 0.07 to 2.16; I^2^ = 86%;* p* = 0.04), respiratory rates were more stable compared with infants in conventional tub bath group (MD = -2.73; 95%CI -3.43 to -2.03; I^2^ = 0;* p* < 0.01). The crying duration, stress and pain scores of preterm infants in swaddle bath group were lower than those in conventional tub bath group (SMD = -1.64; 95CI -2.47 to -0.82; I^2^ = 91%;* p* < 0.01), (SMD = -2.34; 95%CI -2.78 to -1.91; I^2^ = 0;* p* < 0.01), (SMD = -1.01; 95%CI -1.40 to -0.62; I^2^ = 49%;* p* < 0.01). Egger's test showed no publication bias in body temperature, respiratory rate, oxygen saturation, and crying duration.

**Conclusions:**

Swaddle bath is the best bathing method than conventional tub bath and sponge bath in maintaining the stability of preterm infant’s body temperature, blood oxygen saturation and respiratory rate. In addition, swaddle bath also plays a role in reducing cry duration, stress scores, and pain levels of preterm infant compared with conventional tub bath and sponge bath. However, due to the important heterogeneity in some outcomes, future studies with larger sample size and more appropriately design are needed to conduct before recommendation.

**Trial registration:**

Prospero CRD42022377657

**Supplementary Information:**

The online version contains supplementary material available at 10.1186/s12887-023-04280-y.

## Background

According to the Global Action Agenda, 15 million preterm infants are born every year [1]. Bathing can keep the skin clean and promotes comfort for preterm infants [2]. It poses little risk to healthy fullterm infants, but it is a stressful stimulus for preterm infants [3]. Due to the imperfect development of various systems of premature infants, thin stratum corneum of the skin and less subcutaneous fat, bath can cause the instability of physiological indexes and increase behavioral pressure to preterm infants, which in turn leads to serious complications such as hypoglycemia, apnea, pulmonary hemorrhage, and growth retardation [4, 5]. Thus, the debate on how to bath preterm infant has never stopped at home and abroad.

Several studies have shown [6–9] that compared with daily bathing, bathing every 4 days did not increase the number of bacteria on the skin surface which leads to infection. One study recommended bathe every 4 days instead of everyday for frequent bathing increases the risk of stressful stimulation and hypothermia in preterm infants [6]. In some developed countries, some skin protectants are used for preterm infants after bathing. However, the skin of preterm infants is delicate, especially the skin of the external genitalia is thinner. The use of skin protectants increases the risk of skin infection, and then lead to sepsis, especially in infants born weight less than 750 g [10]. Thus, the use of skin protectants after bathing is not recommended [7]. Daily bathing causes some damage to the skin of preterm infants [8], and increases the probability of skin-acquired bacterial colonization. Most scholars believe that high-frequency use of soap is harmful to premature infants, and will make the skin of premature infants drying [9, 11, 12]. It is generally believed that the infants’ skin is acidic during the first week of life [13–16], which can reduce the bacterial colonization of the skin surface and increase the barrier function of the skin [17]. Bathing may change the pH value of the skin. A Brazilian study [18] compared bathing with water and pH-neutral soap found that both reduced the bacterial load on the skin surface, but the effect on sepsis was unclear.

The American Association for Women’s Health, Obstetrics, and Neonatal Nursing and the World Health Organization guidelines have specified the time for the first bath of newborns [19, 20], but did not give the recommendations for the best way to bathe. In 2018, Daniel et al. [21] conducted a systematic review on bathing preterm infants from 1996 ~ 2013. The literature included in this study was published earlier, and only qualitative description was conducted. In recent years, there have been new reported randomized controlled trials. A systematic review [22] in 2021 only conducted a meta-analysis on one outcome indicator "body temperature". The number of included literatures was too small and the network meta-analysis did not yield meaningful results.

## The review

### Objective

The primary objective of this systematic review and meta-analysis was to evaluate the effectiveness of different bathing methods on physiological indexes and behavioral status of preterm infants.

### Search methods

#### Inclusion and exclusion criteria

1. Inclusion criteria: (a) Research type: randomized controlled trials (RCT) on bath methods of preterm infants published in Chinese or English; (b) Population: preterm infants born before 37 weeks of gestation; (c) Interventions: effects of different bathing methods on premature infants; (d) Comparator group: one of the three bath methods (sponge bath, conventional tub bath, swaddle bath) in experimental group, the other of the three bathing methods in control group; (e) Outcomes: (1) the primary outcome was to assess the effectiveness of different bathing methods about physiological indexes including body temperature, blood oxygen saturation, heart rate, respiratory rate; (2) the second outcome was to evaluate the bathing methods on behavioral indicators including crying duration, pain scores and stress scores.
2. Exclusion criteria: (a) Conference papers, abstracts and other documents that cannot obtain original data; (b) Duplicate publications.

#### Search strategy

We conducted a comprehensive search including PubMed, The Cochrane Library, CINAHL, Embase, Web of Science, CNKI, Wan-Fang Database, Sino Med, which covered the period from establishment of the database to February 2023. The subject headings (Mesh) combined with free words, supplemented by manual retrieval and literature retrospective method to systematically retrieve literature. English search strategy as follows: ("infant, premature"[Mesh Terms] OR ("premature infant*"[Title/Abstract] OR "preterm infant*"[Title/Abstract] OR "neonatal prematurity"[ Title/Abstract]) OR ("extremely premature infant*"[Title/Abstract] OR "extremely preterm infant*"[Title/Abstract])) AND ("bath*"[ Mesh Terms] OR "sponge bath*"[Title/Abstract] OR "swaddled bath*"[Title/Abstract] OR ("tube"[All Fields] AND "bath*"[Title/Abstract])).

### Data extraction

According to the tool of Cochrane Handbook for Systematic Reviews of Interventions, two researchers searched the databases independently, imported all retrieved literatures into Endnote X9 software for deduplication, and then conduct the first round of screening by reading the title and abstract and further read the full text for the second round of screening. In case of disagreement, a third researcher adjudicated. Two authors used a predesigned spreadsheet to extract literatures’ characteristics and outcome data independently and then compared for consistency after completion. Data extracted from the final included literatures including: authors, country, year of publication, type of study design, participant characteristics, sample size, intervention measures, and outcome indexes.

### Quality appraisal

According to the Cochrane Risk of Bias tool for randomized controlled trails (RoB 2) [23], two researchers assessed the quality of the included studies. If the two researchers disagree, the third researcher will decide. The quality evaluation includes the following seven aspects: random sequence, allocation concealment, blinding of study participants and outcome assessors, completeness of outcome data, selective reporting bias and other biases. The literature fully met the above criteria are considered as low bias and quality evaluation is grade A; if partially met, it is moderately biased, and the quality evaluation is grade B; if completely inconsistent, it is highly biased, and the quality evaluation is grade C. Grade A and B were included in this study.

### Date synthesis

Reman Version 5.4 statistical software was used for Meta-analysis. First, the heterogeneity of the literature was tested. If *p* < 0.1 or I^2^ > 50%, it indicated that the heterogeneity among the included literature was obvious. The random effect model was used to analyze. The sensitivity analysis was carried out by excluding the literature one by one to judge the stability of Meta-analysis results. Subgroup analysis was performed on outcome indicators with large heterogeneity to find the source of heterogeneity; If *p* ≥ 0.1 and I^2^ ≤ 50%, it indicated that no significant heterogeneity among the included literatures, and the fixed effect model was used for analysis. All data in this study are measurement data and the mean Difference (MD) is used as the effect indicator. If the measurement units are inconsistent, the standardized mean difference (SMD) is used as the effect indicator. Each effect size estimated using a 95% confidence interval. *p* < 0.05 means the difference is statistically significant. Stata 16.0 software was used to conduct Egger's test on body temperature, respiratory rate, blood oxygen saturation and crying duration, and *P* < 0.05 was considered as publication bias.

### Subgroup analysis

Subgroup analysis was conducted by researchers to further explore the causes of the significant heterogeneity.

## Results

### Characteristics of the included studies

**Fig. 1:**
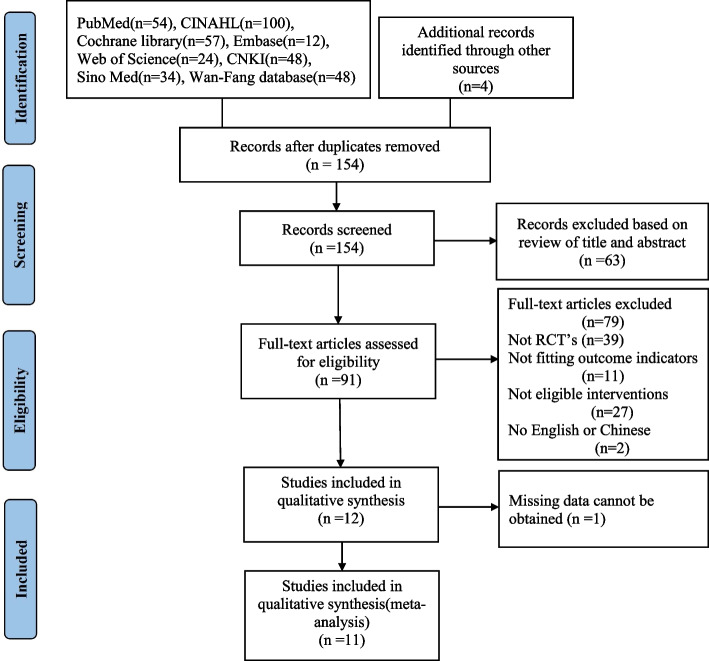
PRISMA Flow diagram

The study selection process is illustrated by the PRISMA Study Flow Diagram (see Fig. 1). A preliminary search obtained 381 literatures, 227 duplicated studies were removed by using EndNote software. Sixty-three articles were excluded due to the titles and abstracts that were not relevant to this study. Two researchers read the full text of 91 literatures respectively, of which 39 articles were not RCT, 2 articles were not published in Chinese or English, 2 articles full texts could not be obtained, 11 articles were not fitting outcome indicators, 27 articles were not eligible interventions. Twelve RCTS were found suitable for inclusion in this study. One article was excluded for cannot get missing data from the original authors. Finally, 11 articles met the inclusion criteria and included in this meta-analysis [3, 24–33].

In this systematic review descriptive, detailed characteristics of the data are presented in Table 1. Overall, 11 included studies were published from 2014 to 2022, including 828 preterm infants. The median gestational age of preterm infants ranged from 30.8 to 36.1 weeks, while the median birth weights varied from 1509.60 to 2899.83 g. Three studies were conducted in Iran [24–26], three in Turkey [27–29], two in China [3, 30], one in Brazil [31], one in the United States [32], one in Indonesia [33]. Samples size ranged from 25 to 60, gestational age varied from 30 to 37 weeks. About study design, nine studies were parallel RCTs [3, 24–26, 28–30, 32, 33] while two were crossover RCTs [27, 31]. Eleven studies include three bath methods, including conventional tub bath, sponge bath and swaddle bath. Most of the studies (*n* = 7) comprehensively compared the conventional tub bath with swaddle bath [3, 24–26, 30, 31, 33]. One crossover RCT [27] compared swaddle bath with sponge bath. Three studies [28, 29, 32] compared the conventional tub bath with sponge bath.

**Table 1:** Characteristics of included studies in systematic review

Author, Year	Country	Study Design	Participant Characteristics	Sample size (N)		Outcome Indexes
				**The control group**	**The experimental group**	
Monkhtari 2021 [24]	Iran	Randomized controlled trial	< 37 WGA	Conventional tub bath (40)	Swaddle bath (40)	(8)
Ceylan 2018 [27]	Turkey	Cross randomized controlled trial	33 ~ 37 WGA	Sponge bath (35)	Swaddle bath (35)	(1)(2)(3)(4)(9)(10)(11)
de 2018 [31]	Brazil	Cross randomized controlled trial	32 ~ 36 WGA	Conventional tub bath (43)	Swaddle bath (43)	(1)(2)(4)(6)(12)
Edraki 2014 [25]	Iran	Randomized controlled trial	30 ~ 36 WGA	Conventional tub bath (25)	Swaddle bath (25)	(1)(9)
Huang 2022 [30]	China	Randomized controlled trial	< 37 WGA	Conventional tub bath (30)	Swaddle bath (30)	(1)(2)(3)(4)(9)(11)
Loring 2012 [32]	The US	Randomized controlled trial	35 ~ 36 ^6/7^ WGA	Conventional tub bath (50)	Sponge bath (50)	(1)
Tasdemir 2019 [28]	Turkey	Randomized controlled trial	34 ~ 36 ^6/7^ WGA	Conventional tub bath (60)	Sponge bath (60)	(1)(2)(3)(4)(13)
Dag 2022 [29]	Turkey	Randomized controlled trial	34 ~ 37 WGA	Conventional tub bath (48)	Sponge bath (48)	(1)(2)(3)(4)(5)
Sun 2021 [3]	China	Randomized controlled trial	32 ~ 37 WGA	Conventional tub bath (40)	Swaddle bath (40)	(1)(2)(3)(4)(7)(9)
Tambunan 2022 [33]	Indonesia	Randomized controlled trial	30 ~ 37 WGA	Conventional tub bath (18)	Swaddle bath (18)	(1)(2)(3)(4)(9)(14)
Paran 2016 [26]	Iran	Randomized controlled trial	30 ~ 36 WGA	Conventional tub bath (25)	Swaddle bath (25)	(9)

(1) Body temperature; (2) Heart rate; (3) Respiration rate; (4) Oxygen saturation; (5) Blood pressure; (6) Salivary cortisol; (7) Pain Score (NIPS);(8) Behavioral Stress Score; (9) Crying time; (10) Pain and stress scores (ALPS-Neo); (11) Stress in premature infants (NSS);(12) Sleep–wake behaviors; (13) Pain intensity; (14) Premature Infant Pain (PIPP)

Body temperature was measured by different types of thermometers or skin heat probe, heart rate and respiratory rate were measured by stethoscope or multiparametric monitor sensor attached to infant’s foot, and blood oxygen saturation was also measured by multiparametric monitor sensor attached to infant’s foot too. Crying duration was the total crying time during the bath or the percentage of the bathing time and the data was obtained through video recording or on-site observation. One study [31] measured the infant’s sleep–wake state behaviours by a video camera. The studies measured infants pain using three tools, including Premature Infant Pain Profile (PIPP), ALPS-Neo Pain and stress assessment scale, Neonatal Infant Pain Scale (NIPS). Newborn Stress Scale (NSS) were used to measure infant’s stress and Comfort NEO Scale was used to measure infant’s pain intensity. All preterm infants included in studies had calm and stable condition before bathing, no contraindications to bathing (such as congenital defects, use of muscle relaxants or sedatives, infection, etc.). The bathing was conducted at least 6 h after birth and more than 1 h after milk feeding.

### Risk of bias of included studies

**Fig. 2:**
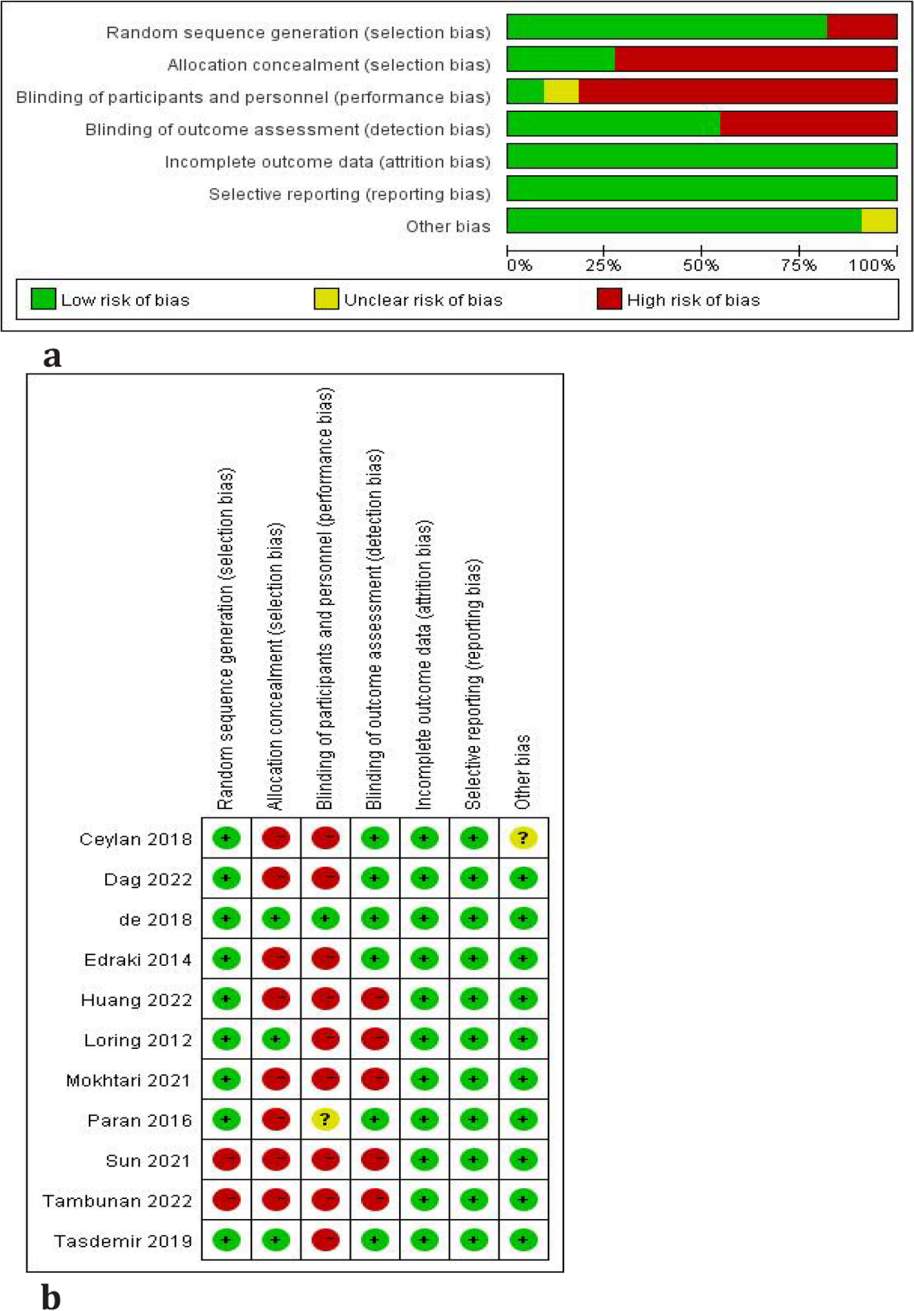
**a** Risk of bias in the included trials. Green indicates a low risk of bias, yellow indicates an unclear risk of bias (inability to evaluate based on reported methodology), and red indicates a high risk of bias. **b** Risk of bias in the included trials. Green indicates a low risk of bias, yellow indicates an unclear risk of bias (inability to evaluate based on reported methodology), and red indicates a high risk of bias

A total of 11 RCTs were included in our study, one was grade A [31] and the remaining 10 were grade B. The evaluation results are shown in Fig. 2a and b. Nine of the 11 studies [24–32] appropriately reported the random -sequence generation while two studies [3, 33] did not clearly describe. Only three studies [28, 31, 32] described their allocation concealment method. Only one study [31] successfully conducted the doubled-blind methods. Six studies [25–29, 31] conducted blind methods to outcome assessment. All studies were in low-risk for incomplete outcome data and selective reporting bias. As for other sources of bias, ten studies [3, 24–26, 28–33] were judged low and one [27] had unclear risk of bias for insufficient evidence provided.

### Synthesis of results (see Table 2)

**Table 2:** Summary of meta-analysis results

Outcomes	Number of included studies	Heterogeneity test		Effect model	Meta-analysis results		*P*-value
		**I** ^**2**^	***P*** **-value**		MD/SMD	95%CI	
**Body temperature(℃/℉)**							
Sponge bath VS Conventional tub bath	3	0	0.68	Fixed	-0.34	-0.56 ~ -0.12	0.003
Swaddle bath VS Conventional tub bath	5	88%	< 0.01	Random	0.18	0.05 ~ 0.30	0.006
**Respiratory Rate(per min)**							
Sponge bath VS Conventional tub bath	2	0	0.58	Fixed	1.23	-0.08 ~ 2.53	0.06
Swaddle bath VS Conventional tub bath	3	0	0.68	Fixed	-2.73	-3.43 ~ -2.03	< 0.001
**Blood oxygen saturation (%)**							
Sponge bath VS Conventional tub bath	2	39%	0.2	Fixed	-0.39	-0.76 ~ -0.02	0.04
Swaddle bath VS Conventional tub bath	4	86%	< 0.01	Random	1.11	0.07 ~ 2.16	0.04
**Heart rate (per min)**							
Sponge bath VS Conventional tub bath	2	61%	0.11	Random	5.90	0.44 ~ 11.35	0.03
Swaddle bath VS Conventional tub bath	4	78%	< 0.01	Random	-5.65	-11.50 ~ 0.21	0.06
**Crying duration (s /%)**							
Swaddle bath VS Conventional tub bath	6	91%	< 0.01	Random	-1.64	-2.47 ~ -0.82	< 0.001
**Stress Score**							
Swaddle bath VS Conventional tub bath	2	0	0.73	Fixed	-2.34	-2.78 ~ -1.91	< 0.001
**Pain Score**							
Swaddle bath VS Conventional tub bath	2	49%	0.16	Fixed	-1.01	-1.40 ~ -0.62	< 0.001

#### Swaddle bath VS conventional tub bath

##### Body temperature

Five studies [3, 25, 30, 31, 33] totalling 312 preterm infants investigated the body temperature of two bath methods. One [25] of the included studies explicitly stated that mercury thermometer was used, one [33] used infrared thermometers, one used digital [31] thermometers, another [30] stated only that thermometers were used, and another [3] mentioned only that temperature was collected by the responsible nurse. Two of them [25, 31] showed the measurement of armpit temperature. Other studies [3, 30, 33] did not specify where the temperature was measured. The body temperature was measured 10 to 15 min after bathing in all the included literatures. There was obvious heterogeneity among the studies (I^2^ = 88%; *p* = 0.006). The result of random-effects model showed that body temperature in swaddle bath group was significantly higher than that in conventional tub bath group (MD = 0.18; 95%CI 0.05 to 0.30).

##### Respiratory rate

Three studies [3, 30, 33] with 176 participants investigated respiratory rate of two bath methods. One of the included studies [30] explicitly indicated that a stopwatch was used, and the other two [3, 33] indicated that measurements were taken either by the responsible nurse or with a relevant tool. The respiratory rate was measured immediately after bathing. The result of fixed-effects model showed that the respiratory rate in swaddle bath group was lower than that in conventional tub bath group (MD = -2.73; 95%CI -3.43 to -2.03) with none heterogeneity (I^2^ = 0;* p* < 0.01).

##### Oxygen saturation

Four studies [3, 30, 31, 33] including 262 participants investigated oxygen saturation of two bath methods. Two studies [30, 33] used pulse oximeters, one [31] fitted a multi-parameter monitor sensor to the feet of preterm infants, and one [3] mentioned only collection by the responsible nurse. The result of random-effects model showed that the oxygen saturation in swaddle bath group was higher than that in conventional tub bath group 

(MD = 1.11; 95%CI 0.07 to 2.16) with high heterogeneity (I^2^ = 86%; *p* = 0.04).

##### Heart rate

Four studies [3, 30, 31, 33] including 262 participants investigated the heart rate of two bath methods. Three studies [30, 31, 33] were measured using stopwatch, stethoscope, and multiparametric monitor sensor attached to the infant’s foot, and one [3] was only reported to have been taken by the responsible nurse. The result of random-effects model showed that there was insignificant effect compared swaddle bath group with conventional tub bath group (MD = -5.65; 95%CI -11.50 to 0.21) with high heterogeneity (I^2^ = 78%; *p* = 0.06).

##### Cry duration

Six studies [3, 24–26, 30, 33] including 356 participants investigated crying duration of two bath methods. Five studies [3, 24, 26, 30, 33] clearly showed the unit of the time was seconds while preterm infants were crying. Only one study [25] showed the percentage of crying time during the bath. The result of random-effects model showed that the crying duration in swaddle bath group was shorter than that in conventional tub bath group (SMD = -1.64; 95%CI -2.47 to -0.82) with high heterogeneity (I^2^ = 91%*, p* < 0.01).

##### Stress score

Two studies [24, 30] including 356 participants reported the stress scores of the two bath methods. The stress measurement in one study [30] was analyzed based on NSS. The other study [24] described stress measurement referred to similar literatures [25, 26] and made necessary changes, but didn’t describe it specifically. The result of fixed-effects model showed that the stress scores in swaddle bath group was lower than that in conventional tub bath (SMD = -2.34; 95%CI -2.78 to -1.91) with low heterogeneity (I^2^ = 0%; *p* < 0.01).

##### Pain scores

Only two studies [3, 33] involving 116 participants investigated the pain scores between two bath methods. NIPS scale was used in one study [3] while PIPP scale was used in the other study [33]. The meta-analysis showed that the pain scores in swaddle bath group was lower than that in conventional tub bath (SMD = -1.01; 95%CI -1.40 to -0.62) with low heterogeneity (I^2^ = 49%; *p* < 0.01).

#### Conventional tub bath VS sponge bath

##### Body temperature

Three studies [28, 29, 32] including 316 infants investigated the body temperature of two bath methods. One study [32] used Fahrenheit as body temperature measuring unit while the other two [28, 29] used Celsius. Standardized mean difference (SMD) was conducted for meta-analysis. The result of fixed-effects model showed that the body temperature in sponge bath group was lower than that in conventional tub bath (SMD = -0.34; 95%CI -0.56 to -0.12) with none heterogeneity (I^2^ = 0%;* p* = 0.003).

##### Respiratory rate

Only two studies [28, 29] involved 216 infants compared the stability of respiratory rate between two bath methods. One study [28] explicitly stated the respiratory rate was counted for one minute, while the other only described that it was recorded by researcher. Two studies both counted respiratory rate for one minute. The result of fixed-effects model showed that there was insignificant difference between sponge bath group and conventional tub bath group (MD = 1.23; 95%CI -0.08 to 2.53) with none heterogeneity (I^2^ = 0%; *p* = 0.06).

##### Oxygen saturation

Two studies [28, 29] including 216 infants investigated the oxygen saturation between two bath methods. One study [29] used a saturation probe to measure oxygen saturation, and the other study [28] measured it based on plus oximetry device. The result of random-effects model showed that the oxygen saturation in conventional tub bath was higher than that in sponge bath group (MD = -0.39; 95%CI -0.76 to -0.02) with low heterogeneity (I^2^ = 39%;* p* = 0.04).

##### Heart rate

Two studies [28, 29] including 216 infants investigated the heart rate between two bath methods. Both studies used pulse oximeters to measure heart rate. The meta-analysis showed that the heart rate in sponge bath group was higher than that in conventional tub bath (MD = 5.90; 95%CI 0.44 to 11.35) with moderate heterogeneity (I^2^ = 61%;* p* = 0.03).

#### Swaddle bath VS sponge bath

One crossover RCT [27] including 35 premature infants who were born at 33–37 weeks gestation age with a birth weight < 1,500 g investigated the effect on infant’s vital signs, oxygen saturation levels, crying time, and level of stress and pain between two bath methods. A monitor probe was used to measure heart rate (per minute) and oxygen saturation (%), an electronic thermometer was used to measure axillary temperature (Celsius). Respiratory rate was measured by observation for one minute. Infants’ bathing was video recorded to evaluate pain and stress behaviors by NSS and ALPS-Neo pain scales. The result showed that swaddle bath had a positive effect on infant’s body temperature, respiratory rate, oxygen saturation level, heart rate. In addition, crying time during sponge bath were longer than that in swaddle bath (*p* = 0.000). Infant’s stress and pain scores in sponge bath group were higher than that in swaddle bath group (*p* < 0.05).

### Subgroup analysis

**Fig. 3:**
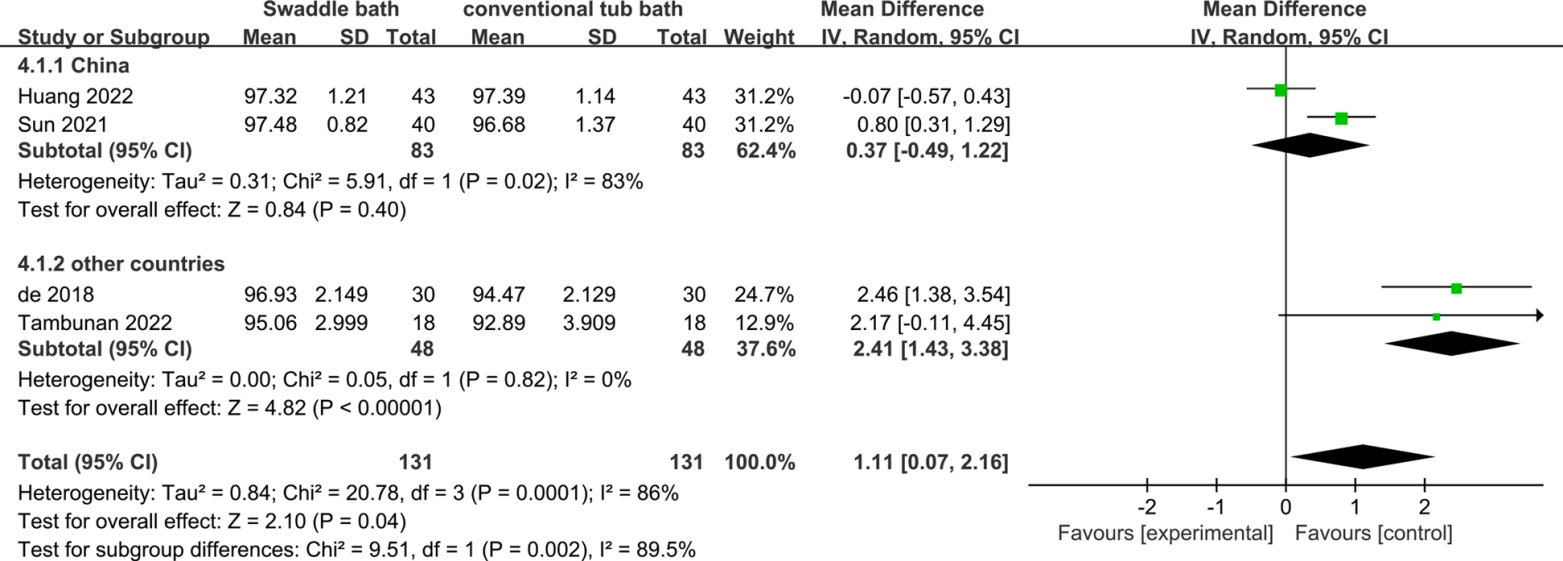
Subgroup analysis of oxygen saturation in swaddle bath (experiment group) vs conventional tub bath (control)

**Fig. 4:**
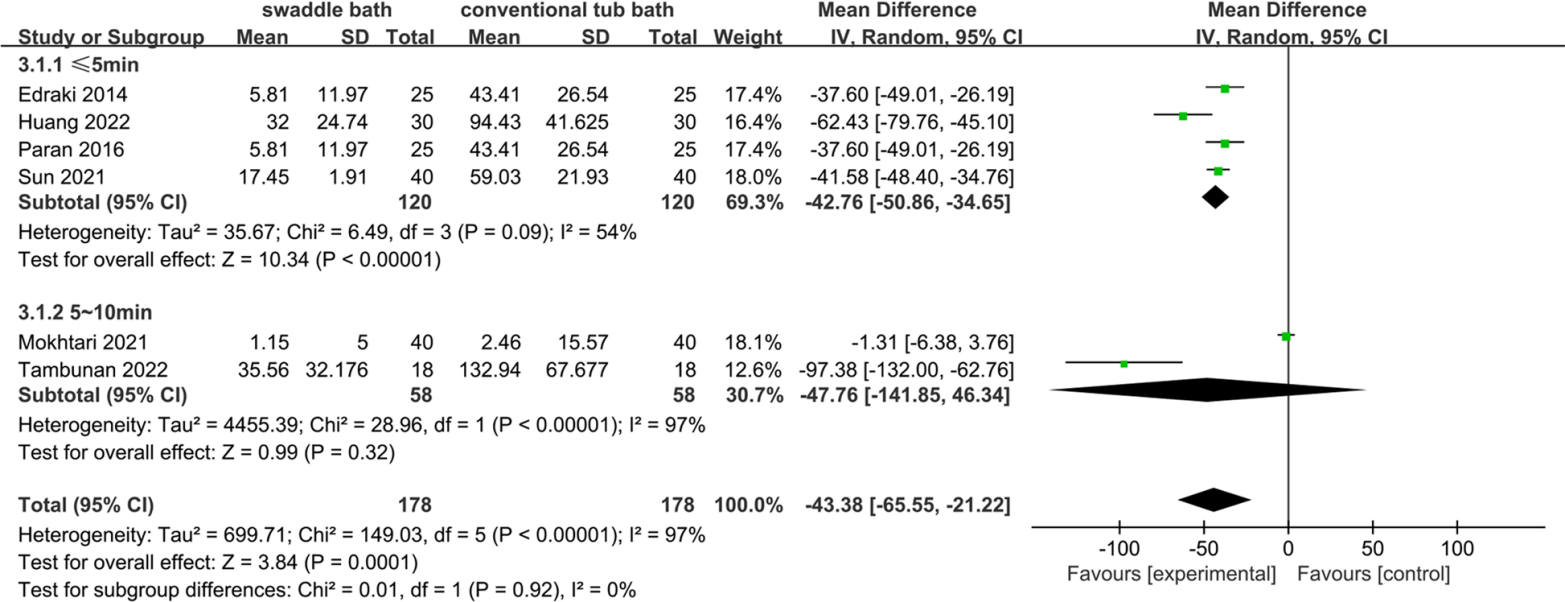
Subgroup analysis of crying duration in swaddle bath (experiment group) vs conventional tub bath (control group)

The heterogeneity of body temperature, blood oxygen saturation and crying duration of preterm infants in swaddle bath and conventional tub bath group was relatively high. Due to limited studies, we didn’t find variables to subgroup the body temperature. As shown in Figs. 3 and 4, subgroup analysis was performed in blood oxygen saturation and crying duration in swaddle bath and conventional tub bath. Ethnic group and regions demonstrated significant effects on oxygen saturation between swaddle bath and conventional tub bath. Subgroup analysis showed that the heterogeneity reduced from 86 to 0%. In addition, bathing duration ≤ 5 min demonstrated significant effects on bathing crying time. Subgroup analysis showed that the heterogeneity reduced from 97 to 54%.

### Publication bias and quality of the evidence

In this study, the result of the Egger's test which included body temperature, respiratory rate, oxygen saturation, and crying duration showed no statistically significant publication bias (*p* > 0.05) (see Table 3). Due to the limited number of studies, Egger’s test was unable to evaluate other outcomes, including heart rate, stress score and pain score.

**Table 3:** Egger's test for publication bias

Outcomes	Group	t	*p*-value	95%CI
Body temperature	Swaddle bath VS Conventional tub bath	-0.19	0.860	-14.00 ~ 12.41
	Sponge bath VS Conventional tub bath	-1.22	0.437	-86.93 ~ 71.68
Respiratory rate	Swaddle bath VS Conventional tub bath	0.03	0.983	-13.84 ~ 13.89
Oxygen saturation	Swaddle bath VS Conventional tub bath	1.36	0.306	-7.51 ~ 14.46
Crying duration	Swaddle bath VS Conventional tub bath	-2.35	0.078	-17.08 ~ 1.41

## Discussion

### Swaddle bath VS conventional tub bath

Compared with the conventional tub bath, the fluctuation of infant’s body temperature, respiratory rate, and oxygen saturation was lower, the crying time was shorter, the stress and pain scores were lower too in swaddle bath. Regarding the infant’s heart rate, there was insignificant difference between two groups. The results of meta-analysis showed that the body temperature and oxygen saturation level of preterm infants in the swaddle bath group was higher than those in conventional tub bath group. The respiratory rate of preterm infants in the swaddle bath group was more stable than that in conventional tub bath group. As for heart rate, there was insignificant difference between two bath groups. Infant’s crying duration was shorter in swaddle bath group compared with conventional tub bath group. Compared with swaddle bath group, infant’s stress and pain scores were higher than those in conventional tub bath group. Bathing is a complex external stress stimuli that can cause physiological instability for preterm infants [34, 35]. Soft towels were used to wrap the whole body of premature infants in a midline position and keep their limbs bend naturally in swaddle bath. As evidences indicated that [36, 37] infants in Swaddling state showed stable vital signs and behavioral status. Infants can relieve pain and sedation through self-regulation, relaxing and promoting them fall asleep as soon as possible [38–40]. In addition, the use of swaddling wraps to put premature infants slowly in water can help relieve stressful experiences caused by sudden environmental changes and reduce crying or pain caused by external environmental stimuli [28]. Therefore, the fluctuations of infant in body temperature, respiration, and oxygen saturation of preterm infants in swaddle bath were smaller than those in conventional tub bath. Moreover, infant’s crying duration was shorter, stress and pain scores were lower in swaddle bath group compared with those in conventional tub bath group.

### Conventional tub bath VS sponge bath

It is concluded from Table 2 that compared with the sponge bath group, the infant’s body temperature, oxygen saturation and heart rate were more stable than those in conventional tub bath group. However, there was no difference in respiration between two groups. Premature infants in NICU often need ventilator support or peripherally inserted central catheterization due to critical condition. Compared with the other two bath methods, sponge bath is easier to implement and is commonly used in NICU [41]. However, one study [42] showed that sponge bath can cause adverse behavioral stress on premature infants because it can make vital signs and behavior in disorders which is not conducive to the growth and development of premature infants. Routine sponge bath is not recommended in preterm infants in guideline [43]. The result of this study also showed that compared with the conventional tub bath, premature infants had a worse tolerance to sponge bath. Infants’ physiological indexes were unstable in sponge bath, including lower body temperature and oxygen saturation, higher heart rates. For preterm infants, bathing is a high stressful nursing practice, which leads to crying, fussing and other adverse emotions [25]. One study [44] had pointed out that conventional tub bath made preterm infants more comfortable compared with sponge bath. Preterm infant immersed in warm water was found quieter and peaceful which reduced heart rate and blood oxygen saturation fluctuations [45]. Moreover, the water covered the infant’s body can reduce evaporative heat dissipation and better maintain the stability of body temperature [44, 45]. This may explain the body temperature, oxygen saturation and heart rate of preterm infants in conventional tub bath are more stable than those in sponge bath.

### Swaddle bath VS conventional tub bath VS sponge bath

After systematic retrieval, only one study compared swaddle bath with sponge bath. Ceylan et al. [27] conducted the trial in preterm infants with gestational age at 33-37 weeks. The preterm infants’ body temperature, respiratory rate level, oxygen saturation level, and heart rates were more stable in swaddle bath group than those in sponge bath group. Swaddle bath also reduce infant’s crying duration, stress scores and pain levels compared to sponge bath. By directly comparing the results of the three bathing methods to preterm infant, swaddle bath is the best bathing method than conventional tub bath and sponge bath in maintaining the stability of infant’s body temperature, oxygen saturation and respiratory rate. Swaddle bath is also better than conventional tub bath and sponge bath in reducing cry duration, stress scores, and pain levels. Conventional tub bath has a positive effect on heart rate and body temperature compared to sponge bath.

### Test of heterogeneity of swaddling bath versus conventional tub bath

The outcome of this study was heterogeneous in body temperature (I^2^ = 88%) and the sensitivity analysis showed stable results by removing study one by one. Due to the limited number of included studies, some original data could not be obtained because of privacy, copyright, and other issues, subgroup analysis for body temperature couldn’t be conducted. It might be related to multiple factors such as different modes of investigators’ operation, gestational age and weight distribution of study participants, age of study participants, and bathing frequency in different included studies. The heterogeneity of oxygen saturation was also high (I^2^ = 86%). The result of subgroup analysis showed that different ethnic groups and regions were one of the sources of high heterogeneity (see Fig. 3). It might be related to the different oxygen-carrying capacity and tolerance to hypoxia of red blood cells in different races. More studies are needed to further verify. The heterogeneity of crying duration was also high (I^2^ = 97%). Subgroup analysis showed that the crying duration of preterm infants was shorter in preterm infants within 5 min of swaddle bathing time (see Fig. 4). There was no difference between two groups if the bathing time was controlled within 5 ~ 10 min. Bathing was a stressful experience for premature infants. As the bathing beginning, preterm infants are put in unfamiliar environment where they are easier to cry. Compared with 5 ~ 10 min bathing duration, bathing controlled within 5 min can make preterm infants get away from stressful stimuli and unfamiliar environments as soon as possible to relaxing and comfort status. The high heterogeneity of some outcomes is not well defined and might have impact on the results. Extra RCT studies with larger sample sizes are needed to find out more causes of the heterogeneity for these three outcomes.

### Limitations

(a) This study is based on published literatures, and not conducted in-depth retrospective of ongoing research or unpublished conference papers or special reports. (b) Some of outcomes in this study were heterogeneous, and no definite source of heterogeneity had been found because some of the original data could not be obtained. (c) The number of included studies in some outcomes are too small that it’s impossible to assess publication bias.

## Conclusion

The results of this meta-analysis indicate that swaddle bath is better than conventional tub bath and sponge baths in maintaining the stability of body temperature, blood oxygen saturation, and respiratory rate in premature infants. Furthermore, Swaddle bath is superior to conventional tub bath and sponge bath in reducing preterm infant’s crying duration, stress scores, and pain levels. These results may exert a positive impact on swaddle bath being recommended as the best bathing method for preterm infants. However, due to the important heterogeneity in some outcomes, further studies focusing on this issue needs to be generated in preterm infants. Randomized clinical trials concentrated on swaddle bath should be encouraged as well.

### Supplementary Information

**Additional file 1:** **Fig. S1.** Meta-analysis of body temperature in swaddle bath vs conventional tub bath. **Fig. S2.** Meta-analysis of body temperature in sponge bath vs conventional tub bath. **Fig. S3.** Meta-analysis of respiratory rate in sponge bath vs conventional tub bath. **Fig. S4.** Meta-analysis of respiratory rate in swaddle bath vs conventional tub bath. **Fig. S5.** Meta-analysis of blood oxygen saturation in swaddle bath vs conventional tub bath. **Fig. S6.** Meta-analysis of blood oxygen saturation in sponge bath vs conventional tub bath. **Fig. S7.** Meta-analysis of heart rate in swaddle bath vs conventional tub bath. **Fig. S8.** Meta-analysis of heart rate in sponge bath vs conventional tub bath. **Fig. S9**. Meta-analysis of crying duration in swaddle bath vs conventional tub bath. **Fig. S10.** Meta-analysis of stress score in swaddle bath vs conventional tub bath. **Fig. S11.** Meta-analysis of pain score in swaddle bath vs conventional tub bath.

## Data Availability

All data generated or analyzed during this study are included in this published article and its supplementary information files.

## Abbreviations

CNKI: China National Knowledge Internet
RCT: Randomized Controlled Trial
NICU: Neonatal Intensive Care Unit
NIPS: Neonatal Infant Pain Scale
NSS: Newborn Stress Scale
WGA: Weeks Gestational Age
PIPP: Premature Infant Pain Profile
MD: Mean Difference
SMD: Standardized Mean Difference
VS: Versus
ALPS-Neo: Astrid Lindgren and Lund Children’s Hospitals Pain and Stress Assessment Scale for Preterm and Sick Newborn Infants
CI: Confidence Interval
Neo: Neonatal
