# Supplementary material for: Effectiveness of different bathing methods on physiological indexes and behavioral status of preterm infants: a systematic review and meta-analysis
Source: BMC Pediatr. 2023 Oct 13;23:507. doi: 10.1186/s12887-023-04280-y (PMC10571243; doi:10.1186/s12887-023-04280-y)
Supplement: Supplementary file 1 — Additional file 1: Fig. S1. Meta-analysis of body temperature in swaddle bath vs conventional tub bath. Fig. S2. Meta-analysis of body temperature in sponge bath vs conventional tub bath. Fig. S3. Meta-analysis of respiratory rate in sponge bath vs conventional tub bath. Fig. S4. Meta-analysis of respiratory rate in swaddle bath vs conventional tub bath. Fig. S5. Meta-analysis of blood oxygen saturation in swaddle bath vs conventional tub bath. Fig. S6. Meta-analysis of blood oxygen saturation in sponge bath vs conventional tub bath. Fig. S7. Meta-analysis of heart rate in swaddle bath vs conventional tub bath. Fig. S8. Meta-analysis of heart rate in sponge bath vs conventional tub bath. Fig. S9. Meta-analysis of crying duration in swaddle bath vs conventional tub bath. Fig. S10. Meta-analysis of stress score in swaddle bath vs conventional tub bath. Fig. S11. Meta-analysis of pain score in swaddle bath vs conventional tub bath. [file 12887_2023_4280_MOESM1_ESM.pdf]

Study or Subgroup	swaddle bath Mean	SD	Total	conventional tub bath Mean	SD	Total	Weight	Mean Difference IV, Random, 95% CI
de 2018	36.54	0.19	43	36.53	0.25	43	20.9%	0.01 [-0.08, 0.10]
Edraki 2014	36.42	0.35	25	35.96	0.26	25	16.5%	0.46 [0.29, 0.63]
Huang 2022	36.85	0.2064	30	36.723	0.2417	30	19.9%	0.13 [0.01, 0.24]
Sun 2021	36.58	0.13	40	36.33	0.13	40	22.6%	0.25 [0.19, 0.31]
Tambunan 2022	36.43	0.1138	18	36.34	0.2064	18	20.1%	0.09 [-0.02, 0.20]
**Total (95% CI)**			**156**			**156**	**100.0%**	**0.18 [0.05, 0.30]**

Heterogeneity: Tau² = 0.02; Chi² = 32.17, df = 4 (P < 0.00001); I² = 88%
Test for overall effect: Z = 2.76 (P = 0.006)

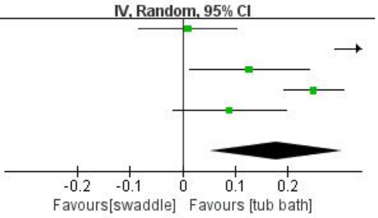

Additional Fig.1. Meta-analysis of body temperature in swaddle bath vs conventional tub bath.

Study or Subgroup	sponge bath Mean	SD	Total	conventional tub bath Mean	SD	Total	Weight	Std. Mean Difference IV, Fixed, 95% CI
Dag 2022	36.52	0.22	48	36.6	0.25	48	30.4%	-0.34 [-0.74, 0.07]
Loring 2012	98.4	0.3576	50	98.6	0.4655	50	31.2%	-0.48 [-0.88, -0.08]
Tasdemir 2019	36.72	0.2	60	36.77	0.22	60	38.3%	-0.24 [-0.60, 0.12]
**Total (95% CI)**			**158**			**158**	**100.0%**	**-0.34 [-0.56, -0.12]**

Heterogeneity: Chi² = 0.78, df = 2 (P = 0.68); I² = 0%
Test for overall effect: Z = 3.02 (P = 0.003)

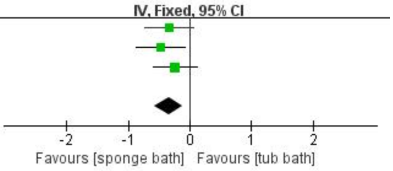

Additional Fig.2. Meta-analysis of body temperature in sponge bath vs conventional tub bath.

Study or Subgroup	sponge bath Mean	SD	Total	conventional tub bath Mean	SD	Total	Weight	Mean Difference IV, Fixed, 95% CI
Dag 2022	55.4	4.93	48	54.51	3.79	48	54.7%	0.89 [-0.87, 2.65]
Tasdemir 2019	47.2	5.41	60	45.57	5.39	60	45.3%	1.63 [-0.30, 3.56]
**Total (95% CI)**			**108**			**108**	**100.0%**	**1.23 [-0.08, 2.53]**

Heterogeneity: Chi² = 0.31, df = 1 (P = 0.58); I² = 0%
Test for overall effect: Z = 1.85 (P = 0.06)

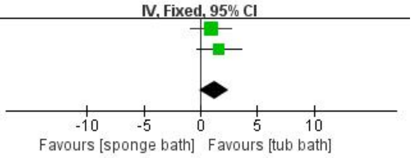

Additional Fig.3. Meta-analysis of respiratory rate in sponge bath vs conventional tub bath.

Study or Subgroup	swaddle bath Mean	SD	Total	conventional tub bath Mean	SD	Total	Weight	Mean Difference IV, Fixed, 95% CI
Huang 2022	43.5	1.306	30	46.17	1.783	30	78.1%	-2.67 [-3.46, -1.88]
Sun 2021	57.59	3.68	40	60.93	4.39	40	15.5%	-3.34 [-5.12, -1.56]
Tambunan 2022	48.33	4.55	18	50.28	3.862	18	6.4%	-1.95 [-4.71, 0.81]
**Total (95% CI)**			**88**			**88**	**100.0%**	**-2.73 [-3.43, -2.03]**

Heterogeneity: Chi² = 0.78, df = 2 (P = 0.68); I² = 0%
Test for overall effect: Z = 7.65 (P < 0.00001)

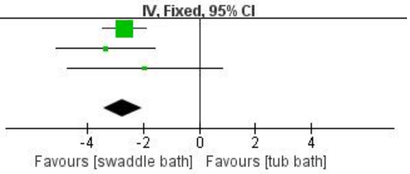

Additional Fig.4. Meta-analysis of respiratory rate in swaddle bath vs conventional tub bath.

Study or Subgroup	swaddle bath Mean	SD	Total	tub bath Mean	SD	Total	Weight	Mean Difference IV, Random, 95% CI
de 2018	96.93	2.149	30	94.47	2.129	30	24.7%	2.46 [1.38, 3.54]
Huang 2022	97.32	1.21	43	97.39	1.14	43	31.2%	-0.07 [-0.57, 0.43]
Sun 2021	97.48	0.82	40	96.68	1.37	40	31.2%	0.80 [0.31, 1.29]
Tambunan 2022	95.06	2.999	18	92.89	3.909	18	12.9%	2.17 [-0.11, 4.45]
**Total (95% CI)**			**131**			**131**	**100.0%**	**1.11 [0.07, 2.16]**

Heterogeneity: Tau² = 0.84; Chi² = 20.78, df = 3 (P = 0.0001); I² = 86%
Test for overall effect: Z = 2.10 (P = 0.04)

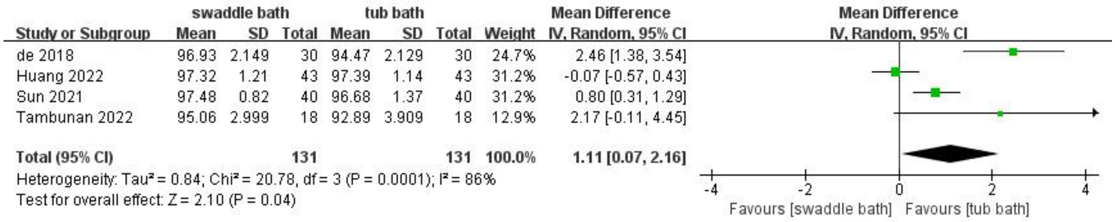

Additional Fig.5. Meta-analysis of blood oxygen saturation in swaddle bath vs conventional tub bath.

Study or Subgroup	sponge bath Mean	SD	Total	conventional tub bath Mean	SD	Total	Weight	Mean Difference IV, Fixed, 95% CI
Dag 2022	95.82	2.3	48	95.68	2.16	48	17.4%	0.14 [-0.75, 1.03]
Tasdemir 2019	97.85	1.36	60	98.35	0.88	60	82.6%	-0.50 [-0.91, -0.09]
**Total (95% CI)**			**108**			**108**	**100.0%**	**-0.39 [-0.76, -0.02]**

Heterogeneity: Chi² = 1.63, df = 1 (P = 0.20); I² = 39%
Test for overall effect: Z = 2.04 (P = 0.04)

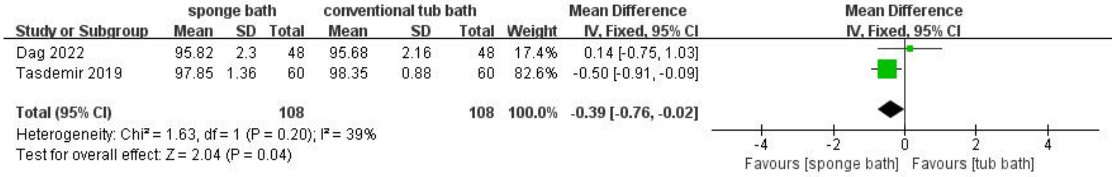

Additional Fig.6. Meta-analysis of blood oxygen saturation in sponge bath vs conventional tub bath.

Study or Subgroup	swaddle bath Mean	SD	Total	conventional tub bath Mean	SD	Total	Weight	Mean Difference IV, Random, 95% CI
de 2018	132.8	10.682	30	143.53	13.452	30	24.1%	-10.73 [-16.88, -4.58]
Huang 2022	132.76	15.43	43	130.29	16.6	43	22.8%	2.47 [-4.30, 9.24]
Sun 2021	143.83	6.54	40	153.8	7.51	40	30.1%	-9.97 [-13.06, -6.88]
Tambunan 2022	114.78	9.137	18	117.5	11.174	18	23.0%	-2.72 [-9.39, 3.95]
**Total (95% CI)**			**131**			**131**	**100.0%**	**-5.65 [-11.50, 0.21]**

Heterogeneity: Tau² = 27.20; Chi² = 13.81, df = 3 (P = 0.003); I² = 78%
Test for overall effect: Z = 1.89 (P = 0.06)

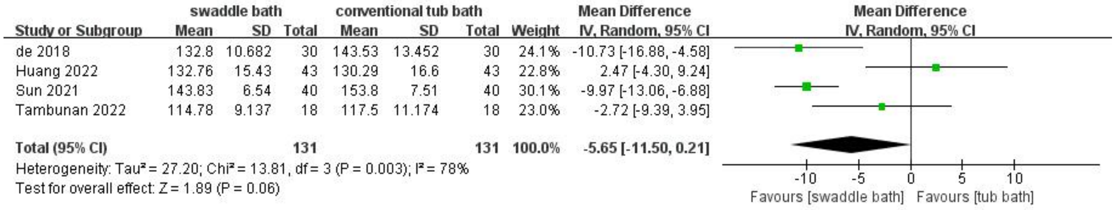

Additional Fig.7. Meta-analysis of heart rate in swaddle bath vs conventional tub bath.

Study or Subgroup	sponge bath Mean	SD	Total	conventional tub bath Mean	SD	Total	Weight	Mean Difference IV, Random, 95% CI
Dag 2022	142	9.72	48	138.57	10.55	48	56.0%	3.43 [-0.63, 7.49]
Tasdemir 2019	139.37	18.95	60	130.33	11.23	60	44.0%	9.04 [3.47, 14.61]
**Total (95% CI)**			**108**			**108**	**100.0%**	**5.90 [0.44, 11.35]**

Heterogeneity: Tau² = 9.55; Chi² = 2.54, df = 1 (P = 0.11); I² = 61%
Test for overall effect: Z = 2.12 (P = 0.03)

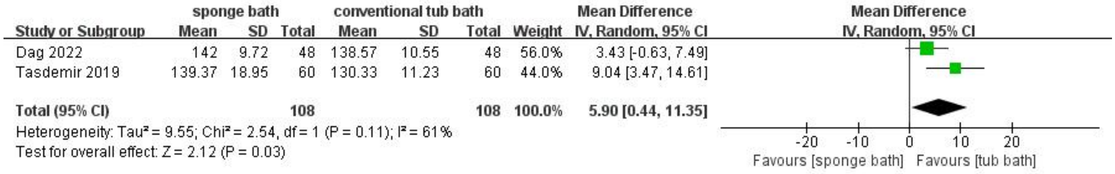

Additional Fig.8. Meta-analysis of heart rate in sponge bath vs conventional tub bath.

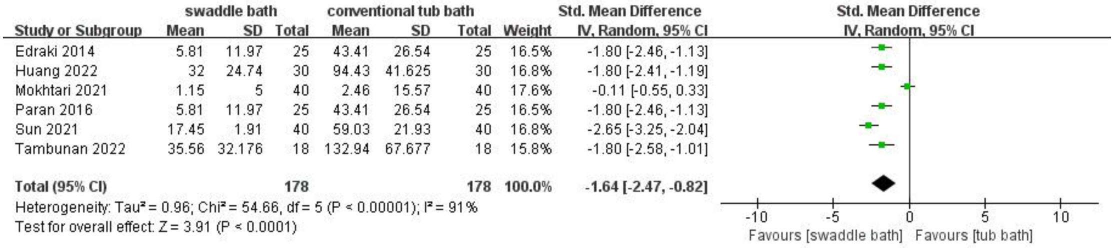

Additional Fig.9. Meta-analysis of crying duration in swaddle bath vs conventional tub bath.

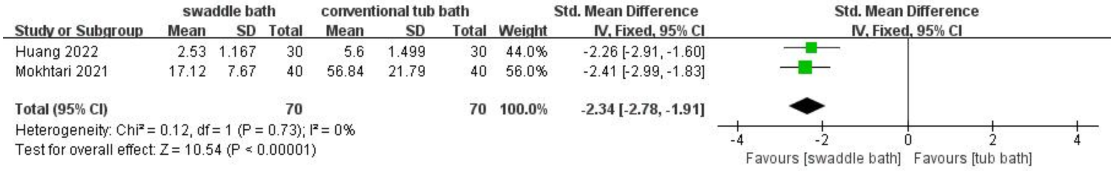

Additional Fig.10. Meta-analysis of stress score in swaddle bath vs conventional tub bath.

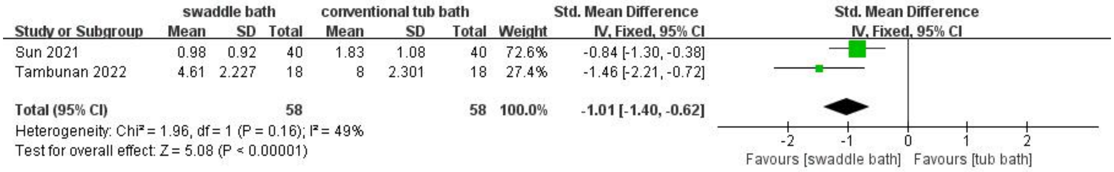

Additional Fig.11. Meta-analysis of pain score in swaddle bath vs conventional tub bath.
